# Research Advances in Natural Polymers for Environmental Remediation

**DOI:** 10.3390/polym17050559

**Published:** 2025-02-20

**Authors:** Muhammad Sheraz, Xiao-Feng Sun, Adeena Siddiqui, Sihai Hu, Zhengcang Song

**Affiliations:** 1 School of Chemistry and Chemical Engineering, Northwestern Polytechnical University, Xi’an 710129, China; sherazmuhammad@mail.nwpu.edu.cn (M.S.); aadeenasiddiqui@gmail.com (A.S.); 2 Shenzhen Research Institute of Northwestern Polytechnical University, Shenzhen 518057, China; 3 Powerchina Northwest Engineering, Xi’an Port Navigation Shipbuilding Technology Corporation Limited, Xi’an 710065, China; songzhengc@nwh.cn

**Keywords:** natural polymers, biodegradability, adsorption mechanism, adsorbents, wastewater treatment

## Abstract

The search for sustainable and efficient remediation techniques is required to control increasing environmental pollution caused by synthetic dyes, heavy metal ions, and other harmful pollutants. From this point of view, natural polymers like chitosan, cellulose, lignin, and pectin have been found highly promising due to their biodegradability, availability, and possibility of chemical functionalization. Natural polymers possess inherent adsorption properties that can be further enhanced by cross-linking and surface activation. This review discusses the main properties, adsorption mechanisms, and functional groups such as hydroxyl, carboxyl, and amino groups responsible for pollutant sequestration. The paper also emphasizes the effectiveness of natural polymers in removing heavy metals and dyes from wastewater and discusses recent advances in polymer modifications, including ionic crosslinking and grafting. This study underlines the ecological potential of natural polymer-based adsorbents in the treatment of wastewater and the protection of the environment as a sustainable solution to pollution challenges.

## 1. Introduction

Water is one of the most critical elements in human life. However, with increasing demand for water, humans face serious challenges related to water quality, which has been degraded by industrial activities, agricultural practices, and urbanization processes. These factors contribute to various environmental degradations that pollute important water bodies like rivers and oceans. This would not only affect the ecosystem badly, but it also jeopardizes human health and the future of humankind [1,2,3,4,5,6,7]. Therefore, there is a pressing need for appropriate, low-cost, accessible, eco-friendly, portable, thermally efficient, and chemically stable technologies and materials to meet the increasing global demand for safe water [8]. Many types of possibly hazardous chemicals are daily applied, transferred, and handled throughout different activities related to households, transportation, and industrial applications. Most of these operations result in incidental spills or other unforeseen accidents that may further result in the release of pollutants into surface and ground waters through direct and indirect paths [9]. The widespread occurrence of these active hazardous chemicals and their persistence in nature has raised serious concern due to their severe ecosystem hazards and harmful impacts on human health [10,11,12]. The most threatening chemicals are those of synthetic organic dyes, heavy metals, oils, and pesticides. Such dyes are being consumed in numerous industries including textiles, tanning, cosmetics, foodstuffs, and pharmaceuticals [13,14,15,16]. Persistence and dissemination of such pollutants in water resources is a key topic of international research. The conventional chemical treatment of wastewater has, in general, some associated environmental drawbacks such as toxicity, high cost, and non-biodegradability.

Natural polymers are biopolymers formed during the growth, development, reproduction, and metabolism of plants, animals, and microorganisms [17,18]. Natural polymers are the solutions for the treatment of wastewater due to sustainability, cost-effectiveness, and environmental friendliness compared with chemical treatment [19,20]. Derived from renewable resources, these polymers are biodegradable and non-toxic, making them the ideal agents for removing waste without long-term environmental damage [21,22]. Natural polymers include chitosan, cellulose, pectin, and lignin, all of which have certain functional groups that allow them to adsorb heavy metals, organic dyes, oils, and other contaminants as well as enable coagulant and flocculation to be applied in removing suspended solids and microorganisms from wastewater [18,23,24,25,26,27]. Using such natural products would mean a more sustainable way of treatment processes on wastewater as well as reducing the need for hazardous chemicals with eventually lower ecological impact of waste. Natural polymer composite-based adsorption techniques have also gained significant attention as an alternative to conventional methods for removing dyes and heavy metal ions in wastewater treatment. Numerous approaches have been suggested to eliminate dangerous dyes and heavy metals from aqua solutions effectively. These include adsorption [28,29,30,31,32], photocatalytic degradation [33,34], flocculation [35,36], coagulation [37], membrane filtration [38], electrochemical processes [39], biosorption [40,41], and many others. Among these methods, adsorption stands out for its very promising capability to remove heavy metal ions, dyes, or other toxic contaminants from wastewater [42].

This review discusses the use of natural polymers such as chitosan, pectin, cellulose, and lignin in wastewater treatment and environmental remediation. Removal of pollutants such as heavy metals, dyes, and oils by the action of adsorption is made possible with the use of these polymers as sustainable solutions due to their eco-friendliness. Their applications in industrial, domestic, and agricultural wastewater treatment show great promise. They are biodegradable, non-toxic, and cost-effective, making them a possible alternative to synthetic materials.

## 2. Adsorption Mechanism

Adsorption is the process where ions or molecules from a gas or liquid adhere to the surface of a liquid or solid. This occurs due to the interactions between adsorbate and the adsorbent [43,44,45,46]. The adsorption mechanism of metal ions and organic compounds onto green adsorbents has been investigated using various theoretical approaches, including kinetic models such as pseudo-first-order kinetics [47,48,49,50,51,52], pseudo-second-order kinetics [53,54,55,56,57,58,59,60], and interparticle diffusion [61,62,63,64]. Additionally, isotherms like Langmuir [65,66,67,68,69,70,71,72], Freundlich [54,73,74,75,76,77,78,79], and Dubinin–Radushkevich (D-R) [80,81,82,83,84,85,86,87] have been explored for deeper understanding.

**Figure 1:**
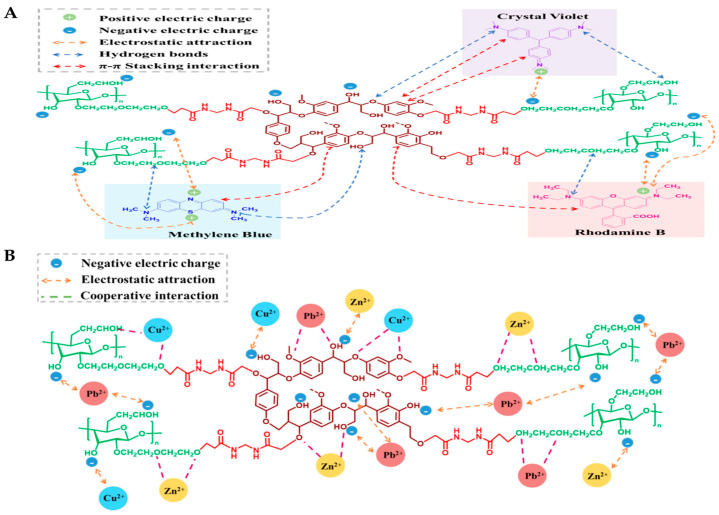
Adsorption mechanism of LCMA for methylene blue, crystal violet, and rhodamine (**A**). Adsorption mechanism of LCMA for Cu^2+^, Pb^2+^, and Zn^2+^ (**B**) [88]. Copyrights © 2025 Elsevier.

Figure 1A demonstrates the adsorption mechanism of lignin/cellulose foam adsorbents (LCMA) for cationic dyes, and Figure 1B describes the adsorption mechanism for heavy metals [88]. The adsorption process by LCMA involves several synergistic processes that allow efficient desorption of cationic dyes and heavy metal ions from water. Electrostatic attraction plays an initial role, considering that the negatively charged functional units in lignin and cellulose attract positively charged pollutants. In addition, stabilization of the adsorption process comes from hydrogen bonding occurring due to the presence of hydroxyl units in the LCMA, attracting pollutants. Also, π-π stacking occurs due to the presence of the aromatic units in the lignin, attracting the dye units and enhancing the bonding capacity. In addition, complexation also plays an essential role during heavy metal ions adsorption, considering that these ions can bond with oxygen-containing functional units, including carboxyl (-COOH) and hydroxyl (-OH) functional units contained in the foam. Finally, the foam’s pore structure invokes the effect of size exclusion, whereby it physically traps bigger units or particles of pollutants, enhancing an additional level of separation. In aggregate, these processes allow efficient, consistent, and efficient performance by LCMA in wastewater treatment.

**Figure 2:**
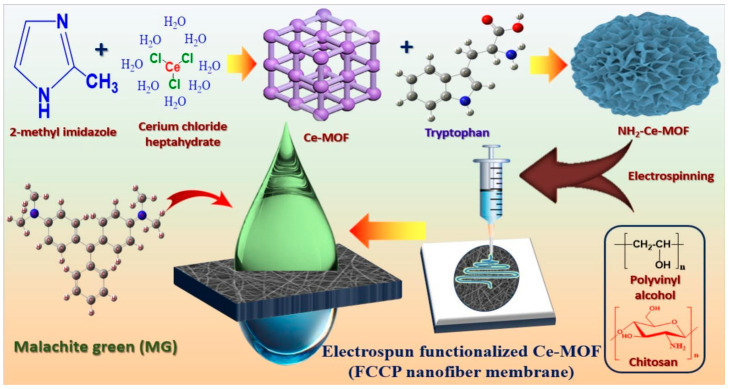
Diagrammatic representation of the FCCP nanofiber membrane production, adsorption, and removal of malachite green (MG) [89]. Copyrights © 2025 Elsevier.

Figure 2 illustrates the diagrammatic representation of the functionalized cerium metal-organic framework/chitosan/polyvinyl alcohol nanofiber membrane (FCCP nanofiber membrane) production, adsorption, and removal of malachite green (MG) [89]. The procedure for the removal of malachite green dye by the FCCP nanofiber membrane involves an intricate adsorption process. Electrostatic forces play an initial role, wherein the positively charged MG dye is attracted onto the negatively charged surface of the FCCP nanofiber membrane. In addition, the extensive surface area and energy of the nanofiber network facilitate physical adsorption, playing an essential role. Besides, π-π interactions between the functional group of the membrane and the dye particles further enhance the efficiency of adsorption. The porosity of the membrane also plays an additional role in the ability of the membrane to adsorb MG dye. These collaborative efforts result in the efficient desorption of the dye, indicating the future scope of FCCP nanofiber membranes in treating wastewater.

**Figure 3:**
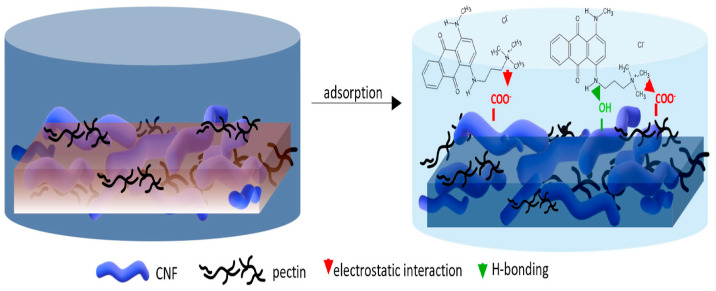
Adsorption mechanism of cation dye BB22 removal by CNF/PC membrane [90]. Copyrights © 2024 MDPI.

Figure 3 proposes the adsorption mechanism of cation dye basic blue 22 (BB22) by cellulose nanofibrils/pectin (CNFs/PC) membrane [90]. The adsorption process of cationic dyes by CNFs/PC membranes is largely driven by electrostatic forces, hydrogen bonding, and the inherent physical properties of the membranes, including pore structure, as well as swelling behavior. A positively charged cationic dye moves toward the negatively charged functional groups found on the membranes’ surface, enabling absorption by means of electrostatic forces. Another source of enhanced absorption is the extent of hydrogen bonding among the functional groups present in cellulose nanofibrils, the dyes, and pectin. These hydrophilic membranes swell in the presence of water, providing increased pore volume and surface area for further attachment sites. The synergy of pore characteristics and swelling further increases the overall capacity for efficient adsorption of cationic dyes from aquatic spaces.

## 3. Major Natural Polymers

Commonly used natural polymers in wastewater remediation include chitosan (a polyaminosaccharide), cellulose (composed of β-(1 → 4)-linked D-glucose units with -OH groups), pectin (a methylated ester of polygalacturonic acid), and lignin (a polymer of propyl phenol units) [91]. These natural polymers are very effective due to sites or functional groups such as amino, carboxyl, hydroxyl, etc., which augment their capacity for the adsorption of metal ions and dyes. The efficiency of an adsorbent in removing any pollutant depends closely on the surface area, structure, and functional groups of the adsorbent. Natural polymers often require some modification, making them high surface area or adding another functional group, to enhance the sorption characteristics. Many strategies have been used to improve the adsorption efficiency for metal ions and dyes [92]. Here, we will discuss the above-mentioned natural polymers.

### 3.1. Chitosan

**Figure 4:**
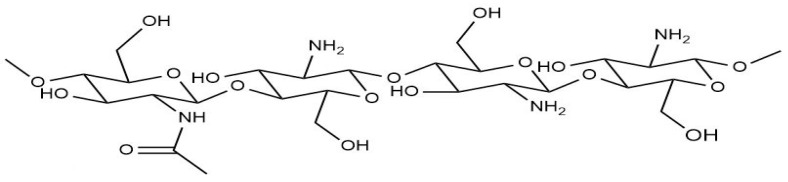
Structure of chitosan.

**Figure 5:**
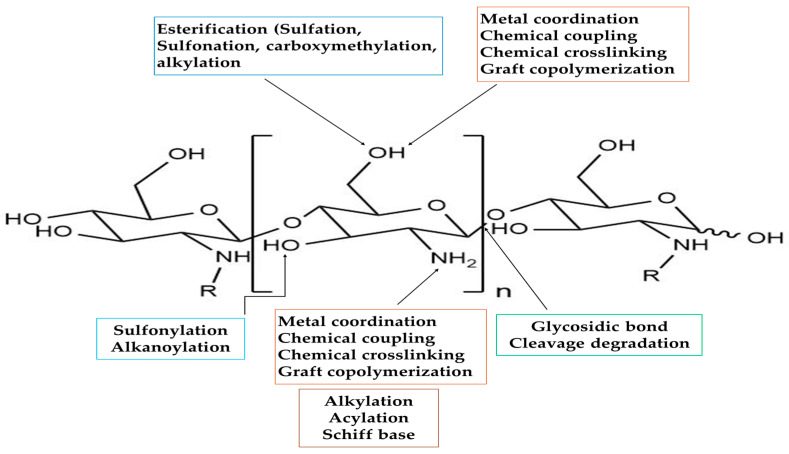
Functional groups of the chitosan structure susceptible to chemical modification.

Chitosan is a linear polyaminosaccharide consisting of β-(1-4)-linked d-glucosamine and N-acetyl-d-glucosamine units. Figure 4 shows the structure of chitosan. It possesses excellent properties such as hydrophilicity, biocompatibility, biodegradability, non-toxicity, and adsorption capability. However, it has pH-dependent solubility [23]. To enhance its chemical and physical activity as an adsorbent, many cross-linking agents were applied, such as hexamethylene diisocyanate, 1-chloro-2,3-epoxypropane [93], polyacrylonitrile [94], tris(hydroxymethyl)-aminomethane, and polyethyleneimine [95]. Figure 5 illustrates the functional groups of the chitosan structure susceptible to chemical alteration.

Adsorption takes place due to the presence of specific interactions between the adsorbent and the adsorbate compound. In the case of chitosan, interactions are closely related to its chemical structure. The main functional groups involved are hydroxyl and amine. Within the pH range 6.0–6.7, the amine groups can be ionized and, hence, pH sensitive. By this ionization, chitosan assumes a polycationic nature and can interact electrostatically with anionic compounds such as certain dyes. The protonated amine groups can attract anionic species by electrostatic attraction or by ion exchange. Besides this, in its nonionized form, one unshared pair of electrons is present in the nitrogen atom; therefore, amine groups can also interact with electron-deficient compounds such as cations. Chitosan can also interact with hydrophilic chemicals, whether anionic, cationic, or neutral. One of the important structural parameters in controlling such interactions is the degree of acetylation. Besides this, by adding an acetyl group, chitosan gains an amphiphilic nature, allowing it to interact with compounds that have slightly hydrophobic properties [96,97]. Organic contaminants pose an environmental challenge in today’s world, with significant concern raised when talking about the removal from aqueous solutions of such kinds of pollutants as dyes, pesticides, herbicides, and pharmaceutical agents. They are persistent in the environment; therefore, their removal becomes a significant concern. Adsorbing chemicals depends on the molecular size and structure as well as polarity, either hydrophilic or hydrophobic traits are considered. Functional groups are also determinants of organic reaction with surfaces of sorbents; this is also true for such interactions that depend on various factors like pH and ion-dipole interactions of involved groups [98].

### 3.2. Cellulose

**Figure 6:**
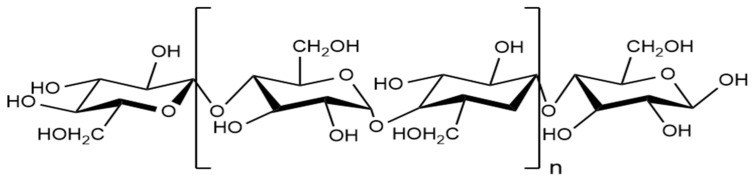
Structure of cellulose.

Cellulose is a biodegradable and non-toxic material that plays a key role in creating sustainable, eco-friendly products [99,100]. It is incredibly versatile and can be found in many sources, like wood (hardwood and softwood), cotton, coir, and bast fibers such as flax, hemp, jute, and rami. It is also present in grasses such as bagasse and bamboo, marine animals such as tunicate, algae, fungi, invertebrates, and bacteria [101,102,103]. Among these, wood is the primary industrial source of cellulose [104,105]. The cellulose polymer chain consists of D-glucopyranose (glucose) molecules connected by β-1,4-glucosidic bonds to form anhydroglucose units. Two anhydroglucose units combine to create anhydrocellobiose, the fundamental repeating unit of the cellulose polymer, as shown in Figure 6 [106].

**Figure 7:**
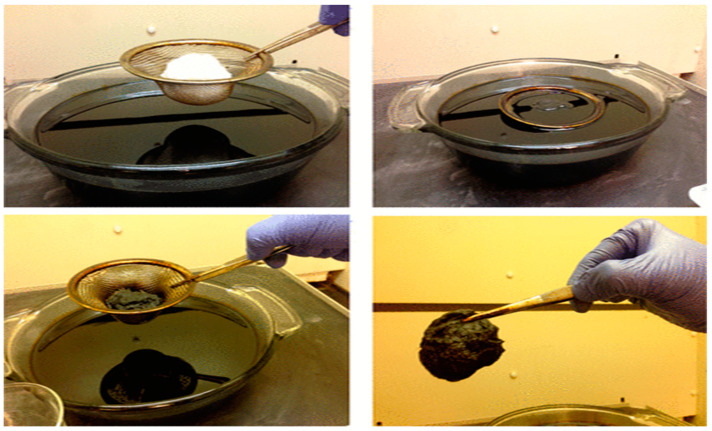
Raw cotton for crude oil uptake [113]. Copyrights © 2013 American Chemical Society.

Functionalizing cellulose through its hydroxyl groups significantly expands its potential use [107,108,109,110,111]. Surface functionalization methods are generally classified into two main categories: (1) chemical modifications, such as TEMPO oxidation and polymer grafting, and (2) physical adsorption, like electrostatic surface adsorption of surfactants. Beyond traditional cellulose fibers, advanced types like microfibrillated cellulose (MFC), with diameters of 10–100 nm and lengths of 0.5–10 mm, and nanofibrillated cellulose (NFC), with diameters of 4–20 nm and lengths of 500–2000 nm, can be created using mechanical techniques such as homogenization and grinding [112]. Raw cotton, a biodegradable sorbent, shows exceptional crude oil adsorption capabilities. Low micronaire cotton adsorbs up to 30.5 g/g of oil, significantly outperforming high adsorption (Figure 7). Analyses reveal a clear link between cotton quality and its sorption performance. Due to its high efficiency and eco-friendly nature, raw cotton is a sustainable and highly effective choice for cleaning up oil spills [113]. A study explored the acetylation of cellulose fibers from corn straw to assess their effectiveness as oil spill sorbents. Under optimal conditions—120 °C for 7 h, the acetylated fibers exhibited impressive oil absorption, soaking up to 90% of diesel oil within just 5 min and aligning with a second-order adsorption model. Characterization techniques confirmed that the modified fibers were oleophilic and water-resistant. These qualities make them an excellent choice for oil cleanup while promoting the sustainable use of agricultural residues [114].

### 3.3. Lignin

**Figure 8:**
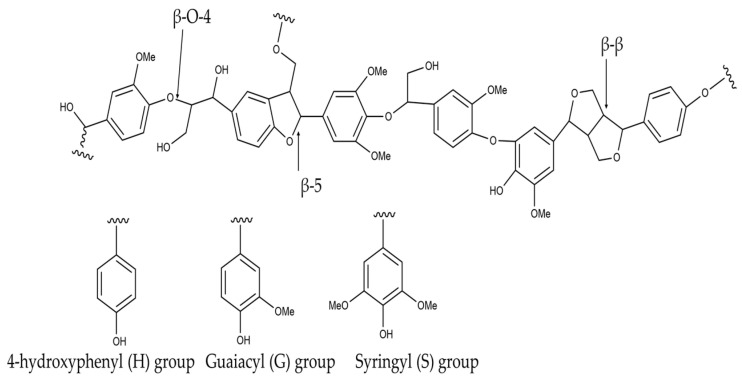
Examples of typical lignin structures showcasing the most common bonds along with the corresponding monomers contributing to its composition, including 4-hydroxyphenyl (H), guaiacyl (G), and syringyl (S) groups.

Lignin is the most abundant aromatic polymer found in nature. It is often a byproduct of industries like paper and pulp production, where it is typically burned as a low-value energy source [115,116,117]. Along with cellulose and hemicellulose, lignin is a key part of wood, wrapping around the cylindrical cellulose fibers within wood cells [118,119,120,121,122]. It is made up of aromatic alcohols—p-coumaryl, coniferyl, and sinapyl—which are categorized by their methoxy group count, enabling strong crosslinking. This gives lignin its highly cross-linked structure, formed through the polymerization of 4-hydroxyphenylpropanoid monomers (monolignols) via ether and carbon–carbon bonds. Lignin’s structure includes phenolic groups like p-hydroxyphenyl (H), guaiacyl (G), and syringyl (S), with their proportions varying depending on the plant species and tissue type. The formation is activated by enzymatic activities of plants that produce enzymes such as peroxidases and laccases, which oxidize monolignols to radicals that cause dimer formation proceeding to polymerization. Most of these couplings occur through the β-carbon and thus might typically include linkages such as β-β, β-O-4, and β-5 bonds, as presented in Figure 8; this means that in structural terms, lignin is indeed an interesting and versatile natural polymer [118,123,124,125].

**Figure 9:**
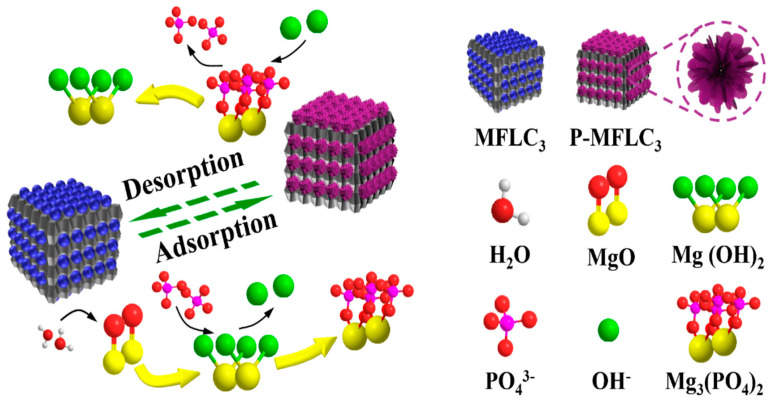
Process for phosphate adsorption/desorption on MFLC_3_ [132]. Copyrights © 2021, Elsevier. During phosphate adsorption, MgO nanoparticles supported on MFLC_3_ firstly turned into Mg(OH)_2_ crystal through bonding with water molecules. These generated hydroxyl ions would be then replaced by phosphates from solution via the ligand exchange. The phosphates could be adsorbed on the MFLC_3_ by forming various Mg_3_(PO_4_)_2_ crystals. During phosphate desorption, these Mg_3_(PO_4_)_2_ crystals on adsorbed-MFLC_3_ could be transformed into Mg(OH)_2_ crystal again in the alkaline solution through an opposite ligand exchange process. Therefore, the adsorbent could be regenerated and reused for the next adsorption process.

Some of the problems of natural lignin are narrow application fields and rather low adsorption capacity. To make it useful in several other fields, scientists usually do some structural modification of the lignin through some chemical treatments. The modification utilizes functional groups in the lignin so that oxidations, sulfonation, carboxylation, alkylation, and amination may occur. It can also be combined with other polymers to create copolymers [126,127,128]. The main mechanism of lignin adsorption is primarily by two routes: physical adsorption of the pollutants through porous structure and ion exchange and chelation of heavy metal ions with hydroxyl, carbonyl, and methoxyl groups of the molecular structure of lignin [129]. In such cases, unmodified lignin is not very effective for the removal of pollutants in water. Therefore, researchers modify lignin or make some composite lignin to enhance its efficiency while cleaning up the pollutants [130,131]. Single-layer chemisorption characterizes the phosphate adsorption/desorption mechanism onto the MgO-functionalized lignin-based bio-charcoal (MFLC). The most important step in the adsorption is the ligand exchange process, in which phosphate ions replace the surface ligands on MgO nanoparticles. It promises satisfactory efficiency and reversibility of phosphate binding and is conducive to its excellent adsorption performance and regeneration ability of MFLC. Figure 9 illustrates the process of adsorption/desorption by MFLC_3_ [132].

### 3.4. Pectin

**Figure 10:**
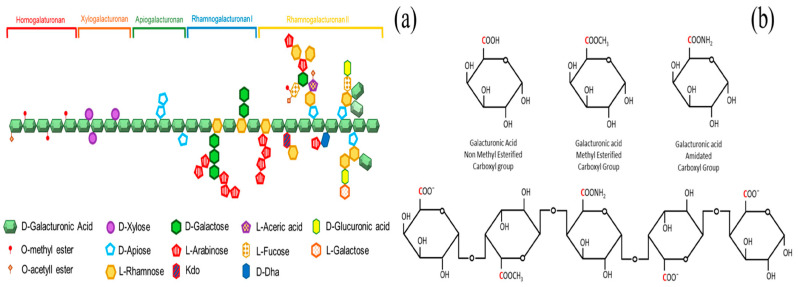
(**a**) Pectin is composed of many different polysaccharides such as homogalacturonan (HG), with other parts including xylogalacturonan, apiogalacturonan, and rhamnogalacturonan II and I (RG-II and RG-I). (**b**) A schematic illustration of a particular structural representation of poly(galacturonic acid) chain with regard to Carbon 6 on the galacturonic acid units and their various functional groups [133]. Copyrights © 2023, MDPI.

Pectin, a natural anionic heteropolysaccharide found in plant cell walls, is especially abundant in fruit peels like citrus, apple pomace, passion fruit rind, pomelo, and banana peels [133]. Its structure is based on α-(1,4)-linked D-galacturonic acid (GalA) residues, which are primarily found in homogalacturonan (HG, about 60%), rhamnogalacturonan I (RG-I), and rhamnogalacturonan II (RG-II) domains. HG is the simpler and most abundant domain in the pectin, which also contains branched regions, called xylogalacturonan, containing sugars such as xylose, apiose, rhamnose, and galactose (Figure 10a). Chemically, GalA residues can be modified at carbon 6 by methyl-esterification (COOCH_3_), carboxylation (COO^−^), or acetylation at the O-2 or O-3 positions, which modifies the degree of esterification (Figure 10b) [134].

Since pectin is a naturally water-soluble polysaccharide, for such a polymer to work effectively as an adsorbent, it must be transformed into an insoluble form. In general, this transformation takes place by means of ionic crosslinking with monovalent (e.g., Na^+^, K^+^), divalent (e.g., Ca^2+^, Cu^2+^, Ni^2+^, Mg^2+^), or trivalent cations (e.g., Al^3+^, Fe^3+^). Non-ionic cross-linkers such as glutaraldehyde or laccase may be employed; however, ionic crosslinking remains the most used method once it presents higher efficiency for the release of contaminants and allows the production of reusable adsorbents for expensive pollutants [135,136]. Traditional methods for the removal of synthetic dyes and heavy metal ions often end in failure. Hence, adsorption has become one of the most suitable alternatives for extracting these contaminants from polluted water. Pectin and its derivatives have proved valuable in this regard. Covalent cross-linking of pectin with adipic acid created cross-linked carboxyl groups and increased the amount greatly in relation to the absorption of heavy metals. The modified pectin was able to reach loading capacities of 1.82 mmol/g for Pb^2+^, 1.794 mmol/g for Cu^2+^, and 0.964 mmol/g for Zn^2+^, which means it could be more useful as a heavy metal contaminant remover from wastewater [137,138].

## 4. Natural Polymer-Based Composites

### 4.1. Chitosan Composites

**Figure 11:**
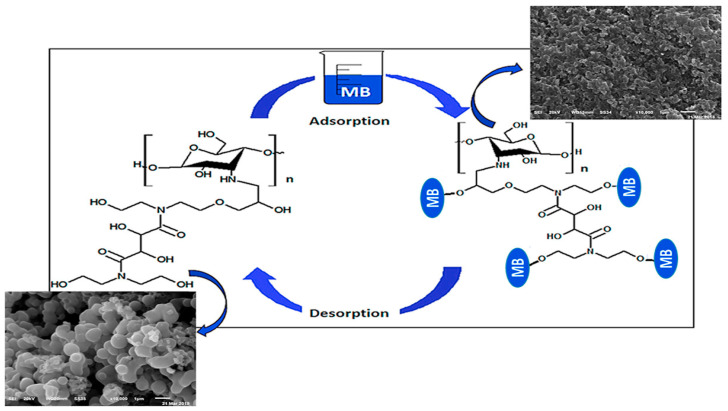
Methylene blue removal by chitosan nano-montmorillonite composites [184]. Copyrights © 2020, Elsevier.

Different materials have been used to synthesize chitosan biocomposites. Examples include magadiite [139], mesoporous silica/nano-γ alumina [140], fly ash-Fe_3_O_4_ [141,142,143], polyaniline [144,145,146,147], montmorillonite [148,149,150], graphene oxide [151,152,153,154,155], epichlorohydrin [156,157,158], diatomaceous earth [159], TiO_2_-P(L-lactide-caprolactone) [160], CeO_2_/Fe_3_O_4_ [161], magnetite [162,163,164,165,166,167,168], salicylaldehyde [92,169,170,171,172,173], vanillin [174], zirconium [175,176,177], banana trunk fibers [178], cellulose nanofibers [179,180,181], and formaldehyde [182,183]. These biocomposites showed high adsorptive capacities in addition to offering improved resistance to acidic media than pure chitosan. Modified chitosan nano-montmorillonite composites were designed in a way that showed efficient removal of methylene blue (MB) up to equilibrium within 8 min, having adsorption capacities ranging between 248.9 mg/g and 276.03 mg/g. Their structure was confirmed by characterization, and adsorptions that occurred followed the Freundlich isotherm and pseudo-second-order kinetics; processes were described as involving electrostatic attraction in alkaline and cation exchange in acidic conditions. High rates of desorption (77.13–97%) were seen in the composites at 0.5 M HCl [184]. Figure 11 shows methylene blue removal by chitosan nano-montmorillonite composites.

### 4.2. Cellulose Composites

**Figure 12:**
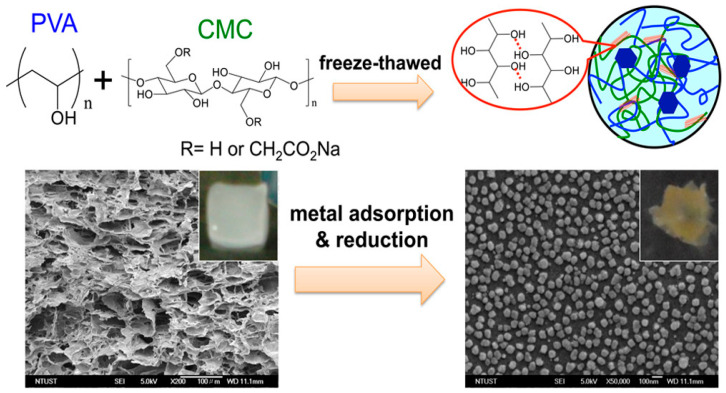
Metal ion adsorption by PVA/CMC hydrogel [213]. Copyrights © 2016 American Chemical Society.

Cellulose composites have been prepared by incorporating various types of materials such as rice husk [185], collagen [186], activated carbon [187,188,189], bisacrylamide [190,191], graphitic carbon nitride (g-C_3_N_4_)/zinc [192], graphene oxide [193,194,195,196], clay [197,198,199,200,201], polyvinyl alcohol (PVA) [202,203], maleic anhydride [204], citric acid [205,206], bentonite [207,208,209,210], and pristine/surfactant [211]. A nanoadsorbent has been synthetized using cellulose nanocrystals (CNCs), which are succinylated to create succinylated CNCs (SCNCs) and sodified to form sodic CNCs (NaSCNCs), to remove lead (Pb^2+^) and cadmium (Cd^2+^). NaSCNCs achieved equilibrium in 5 min and SCNCs in 150 min. Both the adsorbents fitted well with the Langmuir isotherm, with maximum adsorption capacities of 465.1 mg/g and 344.8 mg/g for Pb^2+^ and Cd^2+^, respectively, for NaSCNCs. Both adsorbents showed similar high selectivity, pH-dependent efficiency, and coexisting ion interference resistance [212]. Eco-friendly polyvinyl alcohol (PVA)/carboxymethyl cellulose (CMC) hydrogels were prepared by the freeze-thaw process for metal ion adsorption (Ag^+^, Ni^2+^, Cu^2+^, Zn^2+^) (Figure 12). Hydrogels showed a good swelling ratio along with good selectivity. These hydrogels are good prospects for removing heavy metals from wastewater [213].

**Figure 13:**
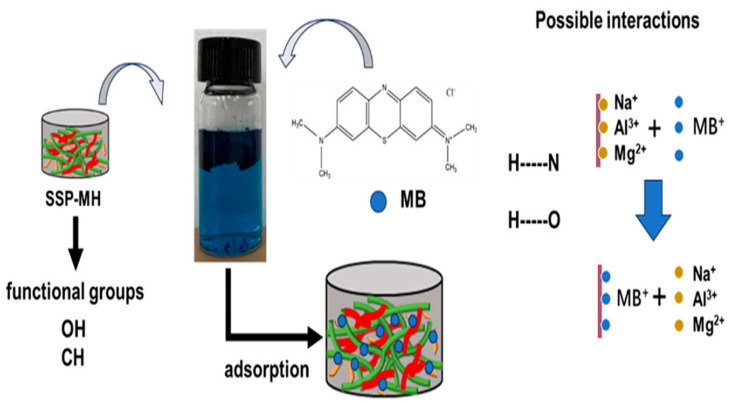
Adsorption mechanism of SSP-MH for methylene blue (blue: MB, green: cellulose, red:1,4-Butanediol diglycidyl ether (BDE), grey: MH) [214]. Copyrights © 2024 Elsevier.

An investigation was conducted to evaluate the synthesis of super adsorbent aerogel (SSP-MH) based on the sunflower stem pith nanocellulose (SSP-C) combined with layered double hydroxides modified montmorillonite (MH) for efficient removal of methylene blue (MB) in the water as proposed in Figure 13 [214]. For the preparation and characterization of SSP-MH, a method has been proposed, examining the increase of its adsorption capacity as compared to pure SSP-C. The removal rate exceeded 87.5% in a solution of MB for this material. The adsorption kinetic tests and thermodynamic analyses confirmed that adsorption performance was well fit to the quasi-second-order adsorption kinetic model and Langmuir isotherm model, proving spontaneous and favorable adsorption. Higher adsorption effectiveness remained with SSP-MH, even after several cycles of testing, promising it to be an economical and reusable biosorbent for waste treatment [214].

### 4.3. Lignin Composites

**Figure 14:**
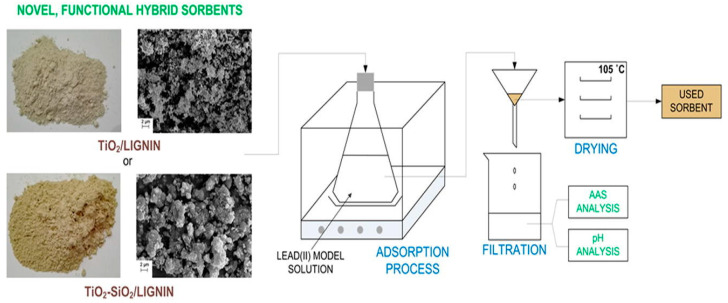
TiO_2_ and TiO_2_-SiO_2_ hybrid lignin-based material for Pb^2+^ removal [232]. Copyrights © 2017, Elsevier.

Lignin composites are prepared by using various types of materials such as polyethylene [215], graphene oxide [216,217,218], bentonite [219], chitin [220], chitosan [221,222,223], Cu/N [224], MnO_2_ [225], amine [226], Fe_3_O_4_ [227], carbon [228], montmorillonite [229,230], MgO [132], and polyoxometalate [231]. A new generation of TiO_2_ and TiO_2_-SiO_2_ hybrid lignin-based materials was synthesized for their abilities to remove Pb^2+^ (Figure 14) [232]. The adsorption capacities were 35.70 mg/g and 59.93 mg/g for TiO_2_/lignin and TiO_2_-SiO_2_/lignin, respectively. The adsorption process followed pseudo-second-order kinetics and Langmuir isotherm with a decrease in adsorption with the increase in temperature at the range of 293–333 K. TiO_2_-SiO_2_/lignin showed much more improved performance and therefore was suitable for Pb^2+^ removal [232]. Eucalyptus Kraft lignin was used to synthesize a hyperbranched polymer for the removal of heavy metals from water. Modified by triazine, oxidized Kraft lignin performed better in terms of absorption, removing 96.1% of Cd^2+^ and 80.4% of Ni^2+^ [233]. A study revealed that a lignin-based adsorbent (LBA) has been modified with 1,2,4-triazole-3-thiol in the format of UV-initiated thiol-yne click reaction during the preparation process to be effectively used for Cd(II). The binding sites in LBA formed by thio-triazole were multiple and thus showed adsorption capacity, which was 8.6 times greater than that of raw lignin. Pseudo-second-order kinetics and Langmuir isotherm corresponded quite well with the adsorption data, with strong selectivity for Cd(II) even in the presence of other competing metal ions. The study demonstrates a straightforward methodology in the preparation of lignin-based materials that possess great potential in heavy metal remediation [234].

**Figure 15:**
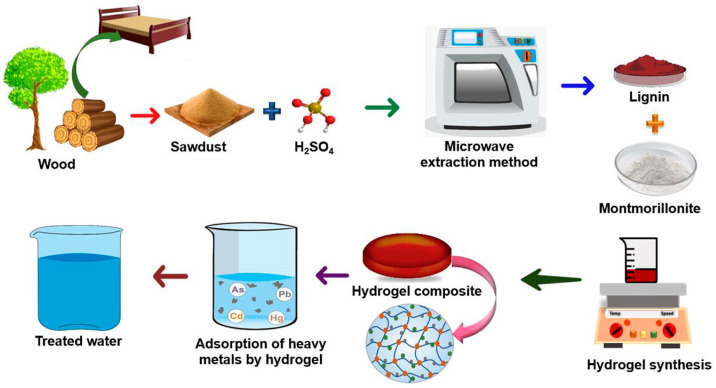
Schematic illustration of lignin–montmorillonite composite hydrogel preparation and adsorption of heavy metals [235]. Copyrights © 2024 American Chemical Society.

A novel lignin–montmorillonite composite hydrogel was investigated for the remediation of water polluted by trace metals [235]. Lignin was extracted from the sawdust of *Tectona grandis* (teak wood) using microwave-assisted acidolysis and combined with montmorillonite for hydrogel formation, exhibiting high adsorption for Pb(II), Cd(II), Hg(II), and As(III) ions. Kinetics and isotherm studies revealed that the adsorption process followed pseudo-second-order kinetics and Langmuir isotherm, indicating favorable monolayer adsorption. The hydrogel also demonstrated excellent reusability with >50% removal efficiencies after five cycles, removing trace metals from actual wastewater samples, thus showing great promise as a sustainable and economically viable choice for large-scale water treatment. The thermodynamic studies indicate that the adsorption process is spontaneous and endothermic in nature. Figure 15 shows the preparation of lignin–montmorillonite composite hydrogel and the adsorption of heavy metals [235].

### 4.4. Pectin Composites

**Figure 16:**
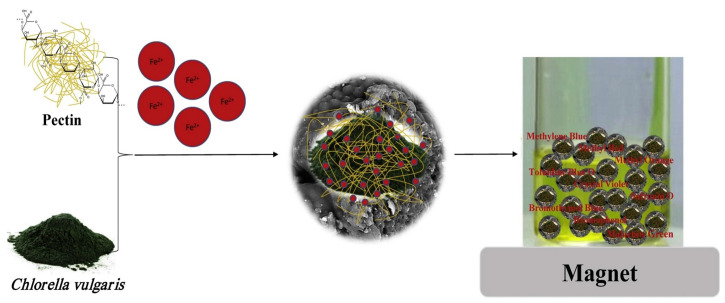
Magnetic pectin–*Chlorella vulgaris*-based biocomposite for various dyes [252]. Copyrights © 2019, Elsevier.

Pectin composites have been synthesized with different substances such as polygalacturonate [236], chitosan [237,238,239], guar gum beads [240], alginate [236,241], cellulose [242,243], montmorillonite [244,245,246], xanthate [247], acacia gum phthalate [248], biochar [249], and others [250,251]. Composite beads were made for multi-metal ions removal using pectin (Pec) and cellulose microfibers (CMF) extracted from orange wastes. Adsorption efficiencies for Cd(II), Cu(II), and Fe(II) fell in the range of 94% to 58%, with maximum capacities of 192.3 mg/g, 88.5 mg/g, and 98.0 mg/g, respectively, and following the order Fe(II) > Cu(II) > Cd(II). FTIR confirmed effective binding sites, and these beads can be reused at least five times, so they are expected to be usefully implemented in wastewater treatment [242]. MPC-50 refers to a synthesized magnetic pectin–*Chlorella vulgaris*-based biocomposite, which is efficient for dye adsorption in wastewaters. This biocomposite contains 50% pectin and 50% *C. vulgaris* and offers an adsorption efficiency of 930 mg/g for various dyes (e.g., methylene blue, methyl orange). Pectin was responsible for efficient adsorption by safeguarding active sites from obstruction by Fe_3_O_4_ and by having interactions with dye molecules. Fe_3_O_4_ ensured its easy separation. It was reusable for many cycles through microwave-assisted regeneration for a sustainable and effective alternative for wastewater treatment (Figure 16) [252].

**Figure 17:**
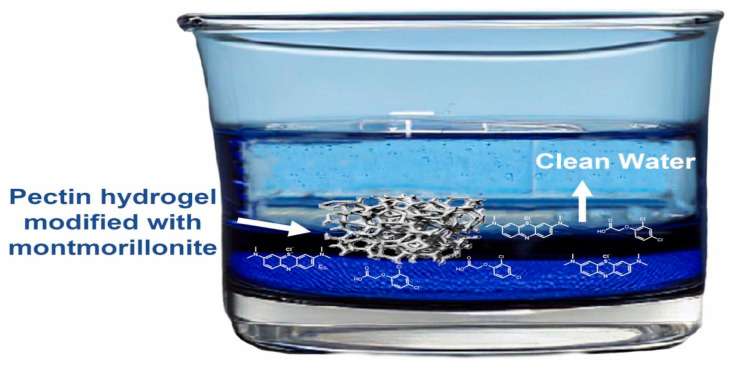
Pectin hydrogel modified with montmorillonite for the removal of MB [253]. Copyrights © 2023, Elsevier.

The application of pectin-based hydrogels modified with montmorillonite (MMT) was studied for the removals of methylene blue (MB) and 2,4-dichlorophenoxyacetic acid (2,4-D) from water as depicted in Figure 17 [253]. The characterization of the hydrogels and the interaction analysis between the pollutants were performed using FTIR, TGA, DSC, and SEM under various conditions including contact time, pH, temperature, and initial pollutant concentration. The results indicated that composite hydrogels containing lower MMT percentages (e.g., 1% MMT) and higher pectin contents were more efficient for pollutant removal. This was justified by sorption behavior that fit well with the Redlich–Peterson and Sips isotherms, indicating multi-site interactions and monolayer formations. The materials proved to be reusable for MB removal during three cycles after recovery with hydrochloric acid or ethanol. All these features highlight the potential of these biocompatible and biodegradable materials for wastewater treatment, particularly for MB removal [253].

## 5. Applications

### 5.1. Adsorption of Metal Ions

Chitosan and its composites have been used to remove metal ions from wastewater under different experimental conditions [254,255,256,257,258,259,260,261,262,263,264,265,266,267,268,269,270,271,272]. A chitosan-modified montmorillonite (CS-MMT2) aerogel was developed for effective adsorption of Cu^2+^ from wastewater. Removal efficiencies reached a maximum of 98.21% with a capacity of 86.95 mg/g and equilibrium in 50 min. Adsorption kinetics were observed to follow the quasi-second-order model and the Langmuir isotherm, with over 85% efficiency maintained even after 7 cycles. This green, low-cost material is promising for water purification on an industrial scale [273]. A hydrophilic hydrogel made from acrylic acid and chitosan has been developed to remove Cu(II) and Pb(II) from wastewater, with an adsorption capacity of 171 mg/g and 192 mg/g, respectively. It conforms to the Langmuir isotherm and to pseudo-second-order kinetics. It is effective in both batch and column methods. This reusable, eco-friendly hydrogel provides a solution to heavy metal contaminations in a sustainable way [274]. Table 1 lists the chitosan-based adsorbents for metal ion removal.

**Figure 18:**
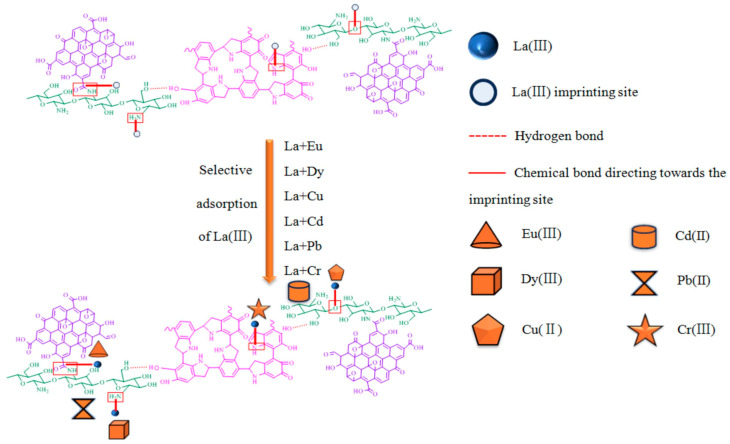
Selective adsorption mechanism of IIP-GO-CS-PDA on La(III) (pale green: CS, purple: GO, pink: PDA) [275]. Copyrights © 2025, Elsevier.

Research has focused on the development of a selective adsorbent for lanthanum (La(III)) recovery from waste using an ion-imprinted graphene oxide–chitosan membrane cross-linked with self-polymerized polydopamine (IIP-GO-CS-PDA) [275]. IIP-GO-CS-PDA was characterized with respect to its structural morphology and thermal stability and was subjected to evaluation for adsorption efficiency and selectivity toward La(III). The material developed a high uptake at pH 5, reaching equilibrium within 30 min and possessing a maximum adsorption capacity of 275.48 mg/g. Furthermore, good selectivity for La(III) over other metals in binary solutions and retention of adsorption capacity after five cycles were observed. This study highlights the potential of such a bio-adsorbent for the selective recovery of La(III) from different sources, as shown in Figure 18.

Cellulose and cellulose composites have been used for adsorption of metal ions for wastewater treatment [276,277,278,279,280,281,282,283,284,285,286,287,288]. Efficiency for Cr(VI) one-step adsorption, reduction, and sequestration, with a value of 386.40 mg/g at a temperature of 25 °C, was achieved using amine-functionalized cellulose aerogel beads (CGP) as adsorbent materials. CGP were found to be stable in different pH conditions and able to show excellent performance in batch and fixed-bed column tests, thus providing a good application opportunity for wastewater treatments [286]. Sulfur-tethered cellulose nanofibers (CNFs) synthesized from medical cotton were investigated for their removal capacity of Pb(II) and Cd(II) from synthetic and industrial waste streams. Characterization of these CNFs showed negative sites for cationic metal ions adsorption. Isotherm batch studies were conducted to determine optimum conditions; the maximum Langmuir capacities were 1.16 and 0.82 mmol/g for Pb(II) and Cd(II), respectively. Regeneration with 0.1 M NaOH retained efficiencies of 90–98% metal removal from industrial waste. The potential of these CNFs has been highlighted and can be expressed as reusable and effective for the remediation of heavy metals [289]. Table 2 lists materials based on cellulose for metal ions removal.

**Figure 19:**
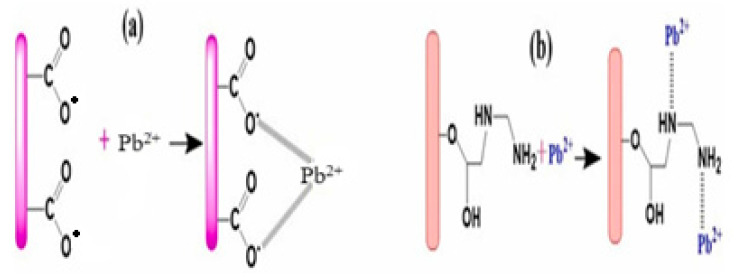
Schematic adsorption interaction of Pb^2+^ with CMC/ADC/MB adsorben: (**a**,**b**) elucidate the main adsorption mechanism through coordination bonds formed between Pb^2+^ and carboxylate or amino group [290]. Copyrights © 2024, Elsevier.

A study evaluated the removal of lead ions (Pb^2+^) from aqueous solutions using a green composite, sodium carboxymethyl cellulose/dicarboxylic acid with modified bentonite composite (CMC/ADC/MB) [290]. The study included the synthesis of the composite and its characterization by using techniques such as FTIR, XRD, FESEM, and TGA, followed by batch adsorption studies to obtain optimum pH, dosage, concentration, and contact time. The results indicated that the maximum adsorption of Pb^2+^ onto this composite was 122.19 mg g^−1^, and the experimental data also fitted the pseudo-second-order kinetics and the Redlich–Peterson isotherm models, suggesting that Pb^2+^ was chemisorbed and immobilized on the surface of the composite in monolayer and showed its ability to remove Pb^2+^ with good reusability. Figure 19 presents the possible adsorption of Pb^2+^ with the adsorbent.

Lignin and its composites have been used to remove metal ions from wastewater [224,291,292,293,294,295,296,297,298,299,300]. Magnetic polyethyleneimine lignin (M-Lignin-PEI) was manufactured for Pb(II) removal. It achieved 96.60 mg/g of adsorption capacity and recorded a removal rate of 99.73%. The adsorbent showed efficient adsorption following the Langmuir isotherm and pseudo-second-order model. This object retained 85% capacity after five cycles of regeneration [301]. A porous lignosulfonate/chitosan adsorbent was synthesized by the radical polymerization of acrylic acid in a lignosulfonate–chitosan solution. The high surface area supplied abundant adsorption sites for heavy metal ions, and the highest removal efficiency was for Cu^2+^ (283 mg/g) and Co^2+^ (386 mg/g). Adsorption equilibrium of 100 mg/L Cu^2+^ was achieved within 60 min using 0.01 g of adsorbent. Heavy metal ion removal was very effective at 0.03 g of adsorbent, which is very cheap and, hence, applicable for practical use in wastewater treatment [302]. Table 3 lists lignin-based composites for the removal of metal ions.

**Figure 20:**
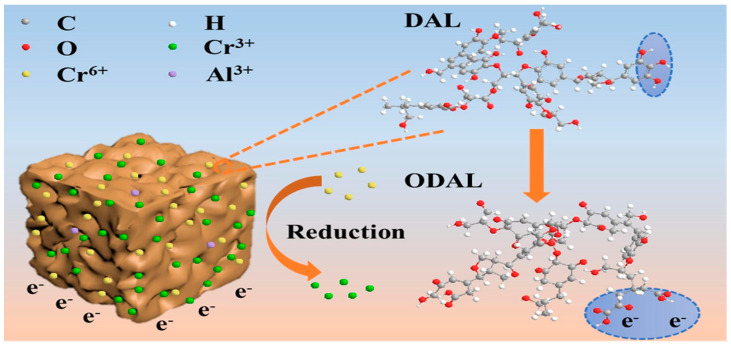
Schematic illustration of DAL adsorption mechanism [303]. Copyrights © 2023, Elsevier.

A study presents a novel approach to wastewater treatment using demethylated lignin (DAL) derived from poplar lignin as an absorbent to efficiently remove Cr(VI) ions [303]. The demethylated lignin had a higher hydroxyl content, and consequently, the resulting DAL adsorbent had a higher adsorption capacity of 703.6 mg/g for Cr(VI) under optimal conditions and reduced Cr(VI) to less toxic Cr(III). In an acidic environment, adsorption of Cr(VI) on the DAL took place through electrostatic attraction with the surface, followed by the reduction by means of phenolic hydroxyl groups, as illustrated in Figure 20. The figure explains the various mechanisms involved in adsorption, in which Cr(VI) is adsorbed on DAL, reduced, and finally retained in the oxidized form of DAL (ODAL), which instills a great deal of confidence in developing an effective, sustainable way of removing Cr(VI) from contaminated water [303].

Pectin and its composites have been prepared to be used in metal ions adsorption for the treatment of wastewater [237,242,304,305,306,307,308,309,310,311,312,313]. One study prepared sodium dodecyl sulfate (SDS)-modified alginate-pectin gel beads (APS221) for the efficient removal of copper ions from water. The APS221 that was prepared through freeze and air drying had a maximum adsorption capacity of 111.11 mg/g. Comparatively, pectin increased metal removal by about 150%, while the addition of sodium dodecyl sulfate improved by 14%. Adsorption was via both complexation and ion exchange, though air-dried outperformed freeze-dried because more ion-exchange sites were retained. The adsorption process followed the Langmuir isotherm and pseudo-second-order kinetics. APS221 was robust, and its efficiency was retained for up to eight cycles, offering a cheap method for the treatment of wastewater [314]. The hydrogel beads made of biochar, pectin, and alginate (BPA) were synthesized for the removal of Cu(II) from grapefruit peel, with maximum adsorption reaching up to 80.6 mg/g at pH 6. BPA was stable and conformed well to the Freundlich isotherm and pseudo-second-order kinetics after five reused runs, while also adsorbing other metals such as Pb(II), demonstrating great potential for application in water treatment processes [249]. Table 4 lists the pectin-based composites for the removal of metal ions.

**Figure 21:**
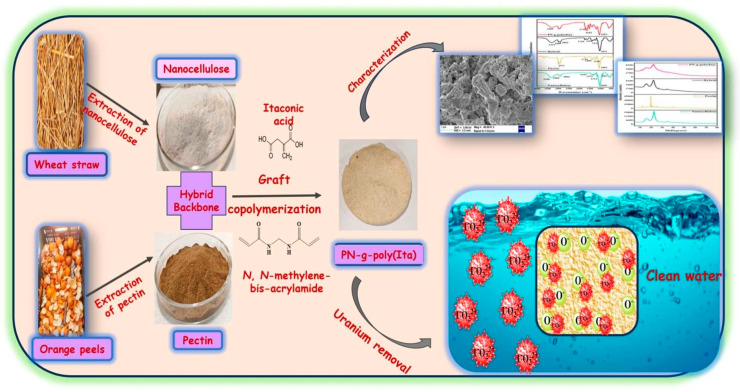
Schematic preparation of PN-g-poly(Ita) adsorbent and the removal of uranium ions [315]. Copyrights © 2025, Elsevier.

Figure 21 shows the schematic preparation of an adsorbent prepared from agricultural wastes (wheat straw and orange peels) and the removal of uranium ions [315]. The emphasis of the research evolved a nanocellulose-pectin graft-poly(itaconic acid)-based hybrid adsorbent (PN-g-poly(Ita)) for uranium removal from water, thus addressing possible environmental hazards while promoting waste-to-wealth strategy. The biosorbent was made by grafting itaconic acid onto a nanocellulose–pectin hybrid backbone, optimizing reaction parameters using response surface methodology (RSM) to attain a grafting percentage of 161.9%. Under optimal conditions (pH 6.0, contact time 210 min, uranium concentration 100 ppb, and adsorbent dose 0.15 g), 97.5% uranium adsorption efficiency had been obtained with the biosorbent, maintaining an 80% efficiency even after six cycles of reuse. The adsorption kinetic followed the pseudo-second-order model, and the equilibrium studies confirmed the Langmuir isotherm with a maximum adsorption capacity of 64.55 µg/g. The investigations present the potentiality of PN-g-poly(Ita) as an environmentally friendly and reusable water remediation alternative that could remove uranium ions and help with environmental management.

### 5.2. Adsorption of Dyes

Chitosan, cellulose, lignin, pectin, and their composites have been used for the adsorption of dyes [190,316,317,318,319,320,321,322,323,324,325,326,327]. A modified ball clay-chitosan composite (MBC-CH) was an efficient adsorbent for the removal of methylene blue (MB), having a capacity of 142 mg/g when used as an adsorbent column in a fixed-bed system. It was efficient at pH levels ranging from 4 to 12 and resistant to salt poisoning, and the adsorption capacity was higher than 50% after five cycles of regeneration. The process was efficient, endothermic, and therefore suitable for the removal of cationic dyes in the water treatment process [328]. Aerogels that are made of cellulosic nanofibrils (CNFs) and graphene nanoplates (GnPs) showed adsorption capacities of at least 1161 mg/g for methylene blue (MB) and 611 mg/g for congo red (CR). It exhibited pseudo-second-order kinetics and Langmuir isotherm model in adsorption. The reusability of the material was demonstrated with ethanol desorption, which achieved nearly 80% dye recovery. Well-suited, these aerogels for wastewater treatment are significant in their potential [329]. A magnetic hybrid material comprising lignosulfonate (FCS) was synthesized to work effectively in dye removal, with maximum adsorption achieved at pH 7 and adsorption capacities of 198.24 mg/g for Congo red and 192.51 mg/g for Titan yellow. It fits well with the Langmuir model, is highly recyclable, and is highly promising for wastewater treatment applications [330]. The adsorption of safranin O dye onto bentonite/acrylamide grafted poly(acrylic acid) hydrogel nanocomposite revealed 97.09% dye removal under optimal conditions. Adsorption took place according to the Freundlich isotherm and pseudo-second-order kinetics, with the assistance of electrostatic interactions, pore filling, and H-bonding. This removal process was found to be spontaneous, exothermic, and efficient for treating real textile wastewater, which provided 95.8% efficiency and stability over five recycle operations [331]. Table 5 lists the removal of dyes by natural polymer composites.

**Figure 22:**
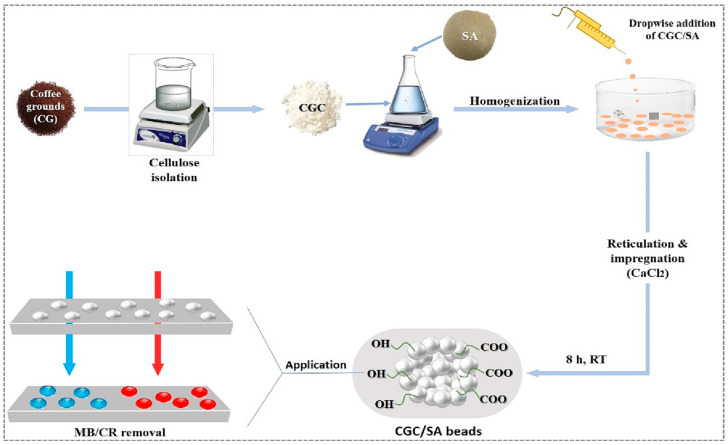
Schematic representation of CGC/SA bead preparation and dye bio-removal process (blue: MB, red: CR) [332]. Copyrights © 2023, Elsevier.

A study investigated the nature and properties of coffee grounds cellulose/sodium alginate (CGC/SA) hydrogel beads as a bio-sorbent viable for removing cationic (methylene blue) and anionic (Congo red) dyes from wastewater (Figure 22) [332]. The beads were developed and characterized with respect to their physicochemical properties, performance, and efficiency appraisals through structural and morphological analyses. Kinetic and thermodynamic assessments revealed rapid equilibrium (20 min) and high adsorption capacities (400.50 mg/g for MB and 411.45 mg/g for CR), best described by the cost-effective Langmuir–Freundlich model. Operational conditions were investigated, including bead mass, contact time, pH, and temperature, which revealed excellent regenerative capability of the beads. The results indicated great prospects for CGC/SA gel beads as bio-adsorbents, which demonstrate excellent adsorption and regeneration capabilities, presenting a valuable and sustainable alternative for wastewater treatment [332].

**Figure 23:**
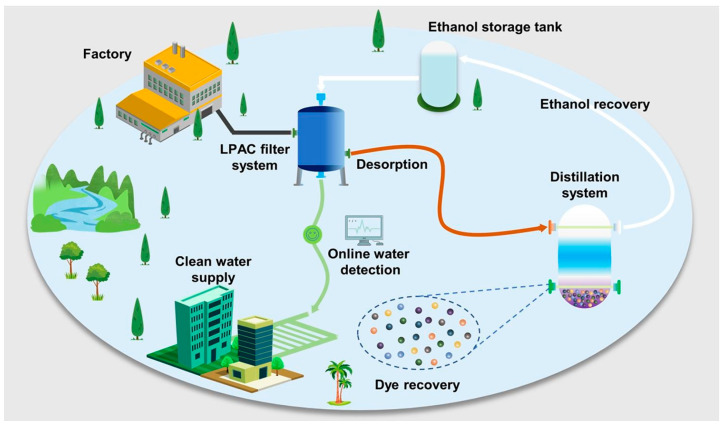
Schematic illustration of the dyeing wastewater treatment [333]. Copyrights © 2024, Elsevier.

A rapid and efficient method has been established for treating dye wastewater with the introduction of a lignin and activated porous carbon (LAPC) filter [333]. Constructed from sustainable lignin material, LAPC features a hierarchically porous structure that promotes water transport and pollutant removal. It has been shown to have good versatility in dye removal and considerable adsorption capacity. The filter is reusable and regenerable and can be subjected to multiple cycles without significantly dropping removal efficiency. The proposed recycling system in a closed loop allows the extraction of clean water, ethanol, and organic dyes, which leads to resource conservation and a sustainable wastewater treatment solution for industries. Figure 23 depicts schematically where the dyeing wastewater treatment and organic dye recovery happen in closed-loop recycling, indicating the high scalability potential for industrial applications along with technology upgrades in wastewater purification [333].

## 6. Challenges and Perspectives

Natural polymers such as chitosan, cellulose, lignin, and pectin offer a viable alternative to traditional wastewater treatment methodologies mainly due to their biodegradability, cost-effectiveness, and availability from renewable resources. However, their full potential remains unfulfilled due to many challenging factors. A significant drawback is that, in most cases, these natural polymers need to be subjected to chemical or physical modifications, such as crosslinking and surface activation, to augment their natural adsorption abilities. Another point to note is that adsorption behavior is highly variable from the type of pollutants targeted, variations in polymer structure and types, and environmental conditions such as pH and temperature. In addition, these adsorptive techniques using natural polymers must show greater efficacy against well-known processes that include photocatalytic degradation, membrane filtration, and electrochemical processes. The need to demonstrate cost-effective solutions is also important. Another challenge concerns scalability; converting and implementing these materials on a large scale for industrial wastewater treatment means that logistical and economic considerations would need to be addressed. Specifically for lignin, shortcomings are imposed by quite narrow application fields and low adsorption capability in its unmodified nature.

Despite this, the prospects for natural polymers in environmental remediation are encouraging. Trend-wise, the emphasis should be laid on composite or new materials prepared by blending natural polymers with other materials to enhance mechanical strength, chemical resistance, and adsorption efficiency. Importantly, the utilization of natural polymers from waste, e.g., agricultural residues, clearly imparts a circular economy value in reducing environmental impact to achieve resource efficiency. Ongoing research is focused on designing tailored materials to target specific pollutants and environmental conditions. Surface modification, regulation of crosslinking, and controlled functionalization provide methods for accomplishing more specific and effective remediation processes. Overcoming limitations in scalability and achieving consistent, high-level performance will pave the way for widespread adoption, solidifying natural polymers as essential tools in tackling global environmental pollution challenges and fostering a more sustainable future.

## 7. Conclusions

In conclusion, natural polymers used in environmental decontamination prove to have substantial potential in combination with inherent limitations. Although modifications are often necessary to improve adsorption capabilities, and performance varies depending on specific conditions, their cost-effectiveness, eco-friendliness, and versatility offer a substantial alternative to conventional approaches. Follow-up research must focus on further development of composite materials, utilization of waste sources as raw materials for their manufacture, and performance improvement to realize their full potential in facing global contamination challenges. Polymer choice is complex and context-dependent, but chitosan and cellulose are often considered the best candidates due to their high pollutant removal capacity for all manners of pollutants, from heavy metals to dyes. Chemical modification by functionalized amino and hydroxyl groups maximizes their utility. Although lignin and pectin have specific benefits to offer, the vast versatility of chitosan and cellulose makes them a popular choice for numerous wastewater treatment processes. Ideally, the determination of the most suitable choice of polymer is subject to specific pollutants of focus and the objectives of the decontamination process.

## Figures and Tables

**Table 1:** Chitosan-based adsorbents for metal ions removal.

Material	Metal Ions	Method	Reference
Chitosan	Co^2+^, Ca^2+^, Cu^2+^, Pr^3+^, Cr^2+^, Eu^2+^, Nd^2+^	Adsorption system	[254]
Chitosan with glutaraldehyde (GLA), epichlorohydrin (ECH), and ethylene glycol diglycidyl ether (EGDE)	Fe^2+^ and Fe^3+^	Batch adsorption system	[255]
Chitosan-graphene oxide	As^3+^, As^5+^	Functionalization	[257]
Fe^o^-nanoparticles-chitosan composite beads	Cr^6+^	Composite preparation	[258]
Fe–Mn binary oxide impregnated chitosan bead	As^3+^, As^5+^	Impregnation	[259]
Iron–chitosan composites	As^3+^, As^5+^	Iron doping and coating	[260]
Chitosan/graphene oxide (CS/GO) membranes with EDTA	Pb^2+^	Blending	[261]
Magnetic chitosan/graphene oxide (MCGO)	Pb^2+^	Ultrasonic dispersion	[262]
Chitosan-grafted polyaniline-OMMT (CPOM) nanocomposite	Cr^6+^, Cd^2+^	Radical polymerization	[263]
Chitosan-g-poly(butyl acrylate)/bentonite nanocomposite	Cr^6+^, Pb^2+^	Ultrasonic-assisted crosslinking	[264]
Glutaraldehyde crosslinked silica gel/chitosan-g-poly(butyl acrylate	Cr^6+^	Sol-gel method	[265]
Chitosan grafted graphene oxide	Cr^6+^	Ultrasonic irradiation technique	[266]
Magnetic chitosan modified with graphene oxide	Cr^6+^	Co-precipitation	[267]
Carboxymethyl chitosan-kaolinite composite hydrogel	Cu^2+^	Schiff base reaction	[268]
1,5-Diphenylcarbazide-chitosan composite hydrogel	Cu^2+^	Diazotization reaction	[269]
Chitosan/Nylon-6 (CS/N) nanofiber membrane	Cu^2+^	Solution blowing technique	[270]
Electrospun chitosan–polyethylene oxide/TEMPO-oxidized cellulose (CS–PEO/TOC) bio-based composite	Cu^2+^	Electrospinning	[271]
Chitosan (CS), waste limestone, and diammonium hydrogen phosphate	Cu^2+^	One-step blending method	[272]

**Table 2:** Cellulose-based adsorbents for metal ions removal.

Material	Metal Ions	Method	Reference
Cellulose acetate nanofibers (CANFs) impregnated with hydroxyapatite (CA/HAp) nanocomposite	Pb^2+^, Fe^3+^	Electrospinning	[276]
Bacterial cellulose/chitosan (BC/CH) composite aerogel	Cu^2+^, Cr^6+^	In situ synthesis, lyophilization	[277]
Thiol-functionalized cellulose nanofiber membrane	Cu^2+^, Cd^2+^, Pb^2+^	Esterification	[278]
Cellulose acetate/citric acid	Cr^6+^	Electrospinning	[279]
Cellulose nanofiber (CNF)/tannic acid/cardanol-derived siloxane aerogel	Cu^2+^	Biomimetic co-deposition, grafting	[280]
Ethylene diamine tetraacetic acid (UiO-66-EDTA)/CNF/CMC aerogel	Cr^3+^, Cu^2+^, Co^2+^, Ni^2+^, Mn^2+^, Zn^2+^, Sn^4+^, Fe^3+^, Zr^4+^	Freeze-drying	[281]
Cellulose ester films (MCC-CAD-GA) based on cellulose (MCC), citric acid anhydride (CAD), and l-glutamic acid (l-GA)	Cd^2+^, Co^2+^, Ni^2+^, Pb^2+^, Cu^2+^	Amidation	[282]
GO/carboxymethyl cellulose nanofibril (CMCNF) composite fiber(CF)	Pb^2+^	Wet-spinning process	[283]
Clay-cellulose biocomposite (CCB)	Pb^2+^, Cd^2+^	Spin and Pressure-Induced Heating	[284]
Polyethyleneimine-crosslinked cellulose	Cu^2+^	Covalent cross-linking	[285]
Cellulose nanofibrils-polyethylenimine-(3-glycidyloxypropyl)trimethoxysilane beads (CGP beads)	Cu^2+^	Cross-linking	[287]
Cellulose nanofiber-sodium alginate hydrogel beads (CNF-SA beads)	Pb^2+^	Cross-linking	[288]

**Table 3:** Lignin-based composites for metal ions removal.

Material	Metal Ions	Method	Reference
Alkaline lignin-graft-poly(acrylic acid) (AL-g-PAA)	Cu^2+^, Cd^2+^	Radical graft copolymerization	[291]
Bentonite/sodium lignosulfonate graft-polymerized with acrylamide and maleic anhydride (BLPAMA)	Pb^2+^, Cu^2+^, Cd^2+^, Zn^2+^	Graft polymerization	[292]
Lignosulfonate-modified graphene hydrogel (LGH)	Cr^6+^	Hydrothermal method	[293]
Chitosan–alkali lignin composite	Cr^6+^	Blending and drying	[294]
Lignosulfonate-modified graphene hydrogel (LS-GH)	Pb^2+^	One-step hydrothermal process	[295]
Porous lignin-based poly (acrylic acid)/organo-montmorillonite nanocomposite	Pb^2+^	Copolymerization, ultrasonic dispersion method	[296]
Lignosulfonate/N-methylaniline composite	Cr^6+^	Cross-linking	[297]
Lignin-branched polyethyleneimine	Cr^6+^	Cross-linking	[298]
Amino-functionalized lignin-based microspheres	Ni^2+^, Cd^2+^, Cr^6+^, As^5+^	Inverse suspension copolymerization	[299]
Lignin particles modified with polyethyleneimine	Cr^6+^	Cross-linking	[300]

**Table 4:** Pectin-based composites for the removal of metal ions.

Material	Metal Ions	Method	Reference
Pectin/γ-Fe_2_O_3_/gl nanocomposite	Cd^2+^, Pb^2+^	Single-step chemical precipitation	[304]
Pectin	Hg^2+^	Response surface methodology	[305]
Modified pectin	Pb^2+^	High hydrostatic pressure	[306]
Pectin/poly(m-phenylenediamine) (P/PmPDA) microspheres	Pb^2+^	Assembly-based synthesis	[307]
Pectin-grafted poly(2-(methacryloyloxyethyl)trimethylammonium chloride-co-2-acrylamido-2-methyl-1-propane sulfonic acid)	Cu^2+^, Pb^2+^, Hg^2+^	Microwave-assisted synthesis, graft copolymerization	[308]
Pectin-based semi-interpenetrating polymer network (semi-IPN) hydrogel	Cu^2+^, Co^2+^, Ni^2+^	Cross-linking	[309]
Chitosan/graphene oxide (CS/GO) membranes with EDTA	Cd^2+^ Pb^2+^, Hg^2+^	Response surface methodology	[310]
Pectin-[(3-acrylamidopropyl) trimethylammonium chloride-co-acrylic acid] hydrogel	Ag^+^	Gamma radiation-induced polymerization	[311]
Pectin-based film blended with chitosan	Cu^2+^	Solvent evaporation method	[312]
Pectin-graphene oxide (Pc/GO) nanocomposite	Cr^3+^	Sol-gel method	[313]

**Table 5:** Removal of dyes by natural polymer composites.

Material	Dyes	Method	Reference
Magnetic chitosan composite microparticles (MCCPs)	Congo red	One-step co-precipitation method	[316]
Chitosan and acid-treated biomass composite(CHI/SFH-SA)	Brilliant green	CHI/SFH-SA precipitation method	[317]
Activated oil palm ash zeolite/chitosan composite	Methylene blue (MB), acid blue 29 (AB29)	Hydrothermal treatment followed by chitosan beading	[318]
Magnetic chitosan-Fe(III) hydrogel	Acid red 73	Chelation procedure	[319]
Cationic polyethylenimine–platinum nanomaterial (PEI-Pt) anchored onto bacterial cellulose (PEI-Pt@BC membrane)	Acid black ATT (anionic dye) and methylene blue (cationic dye)	In-situ reduction	[320]
Amide-functionalized cellulose-based porous adsorbent	Acid black 1, acid red 18 (anionic dyes)	Cross-linking	[190]
Deacetylated cellulose acetate (DA)@polydopamine (PDA) composite nanofiber membrane	Methylene blue	Electrospinning, surface modification	[321]
Sawdust-derived cellulose nanocrystals (CNC) incorporated with zinc oxide (ZnO) nanocomposite	Methylene blue	One-pot ultrasonication, stirring technique	[322]
Fe-modified lignin-based biochar (Fe-LB)	Methylene blue	One-step carbonization method	[323]
Bentonite-doped lignin hydrogel spheres (LHS-BT)	Methylene blue, rhodamine B, malachite green	Template-assisted rapid synthesis method	[324]
Magnetic lignin-based carbon nanoparticles (MLBCN)	Methyl orange	Precipitation-carbonization process	[325]
Pectin modified with a hybrid mixture of *Azadirachta indica* leaves and kaolin clay	Methylene blue, crystal violet	Alkali treatment, precipitation	[326]
Pectin/poly(methacrylic acid-co-2-acrylamido-2-methylpropane sulphonic acid) (pec/poly(MA-co-AMPS)) hydrogel	Methylene blue	Free-radical polymerization	[327]
